# Plant genomic and microbial interplay in the rhizosphere under salt stress: a review

**DOI:** 10.3389/fpls.2025.1667328

**Published:** 2026-01-07

**Authors:** Yi Ren, He Yan, Aiyuan Ma

**Affiliations:** 1 Jiangsu Engineering Research Center for Soil Utilization and Sustainable Agriculture, Jiangsu Center for Collaborative Innovation in Geographical Information Resource Development and Application, Nanjing Normal University, Nanjing, China; 2 Jiangsu Provincial Key Lab of Solid Organic Waste Utilization, Jiangsu Collaborative Innovation Center of Solid Organic Wastes, Nanjing Agricultural University, Nanjing, Jiangsu, China

**Keywords:** rhizosphere microbiome, salt stress, plant–microbe interaction, root exudates, stress resilience

## Abstract

Soil salinization has been considered as a global problem in agriculture, which decreases crop productivity and threatens food security. Salt stress causes complex physiological damages in plants such as ionic imbalance, osmotic stress, and oxidative damage. However, plants have developed several genomic mechanisms to reduce these negative influences that are further supported by dynamic interactions with rhizosphere microbial communities. This review integrates current advances in understanding the interplay between plant genomes and the rhizosphere microbiome under salt stress. It highlights the role of plant-growth-promoting rhizobacteria (PGPR), arbuscular mycorrhizal fungi (AMF), and microbial volatiles in modulating gene expression and root architecture. Notably, PGPR such as *Enterobacter* sp. SA187 and *Bacillus velezensis* have been shown to upregulate key stress-related genes and increase antioxidant enzyme activities, which boost plant resilience under salinity. These microbes also influence stress signaling pathways such as SOS and ABA. Furthermore, this review also discusses the effect of root exudates on microbial communities, the application of synthetic microbial consortia, and genome-scale strategies such as transcriptomics, GWAS, and CRISPR. Our findings show that root exudation patterns shift significantly under salt stress, which enriches beneficial microbial taxa such as *Sphingomonas* and *Streptomyces*, while volatile compounds like benzenoids and ketones contribute to systemic stress responses. Understanding the synergistic plant–microbe interactions provides a foundation to engineer salt-resilient crops and for the advancement of sustainable agricultural practices in saline soils.

## 1 Introduction

Salt stress is one of the most severe abiotic challenges in agriculture. Globally, over 20% of irrigated farmland is estimated to be affected by soil salinization, which significantly limits crop productivity and threatens food security (Atta et al., 2023; Cheng et al., 2025; Rahman et al., 2025). High concentrations of soluble salts cause osmotic imbalance, ion toxicity—particularly from Na^+^ and Cl^−^—and oxidative damage due to excessive reactive oxygen species (ROS) production. These effects disrupt nutrient uptake, impair photosynthesis, and damage cellular structures, which ultimately reduce plant growth and yield. Furthermore, these ions change nutrient balances, block enzyme activity, and disrupt key metabolic pathways (Kumar et al., 2023; Lei et al., 2025).

To cope with these stresses, plants have evolved multiple adaptive mechanisms. These mechanisms are ion homeostasis through selective Na^+^ exclusion and K^+^ retention, osmotic adjustment via the accumulation of compatible solutes such as proline and glycine betaine, and activation of antioxidant defense systems to remove ROS (Abiala, 2025; Liu A. et al., 2025; Liu Q. et al., 2025). A significant part of their resilience is associated with the dynamic interactions between plant genomes and rhizosphere microbial communities (Liu Q. et al., 2022; Oppenheimer-Shaanan et al., 2022). This genomic and microbial cooperation is not only involved in stress responses but also supports primary productivity and nutrient cycling (Adeleke et al., 2024).

**Figure 1:**
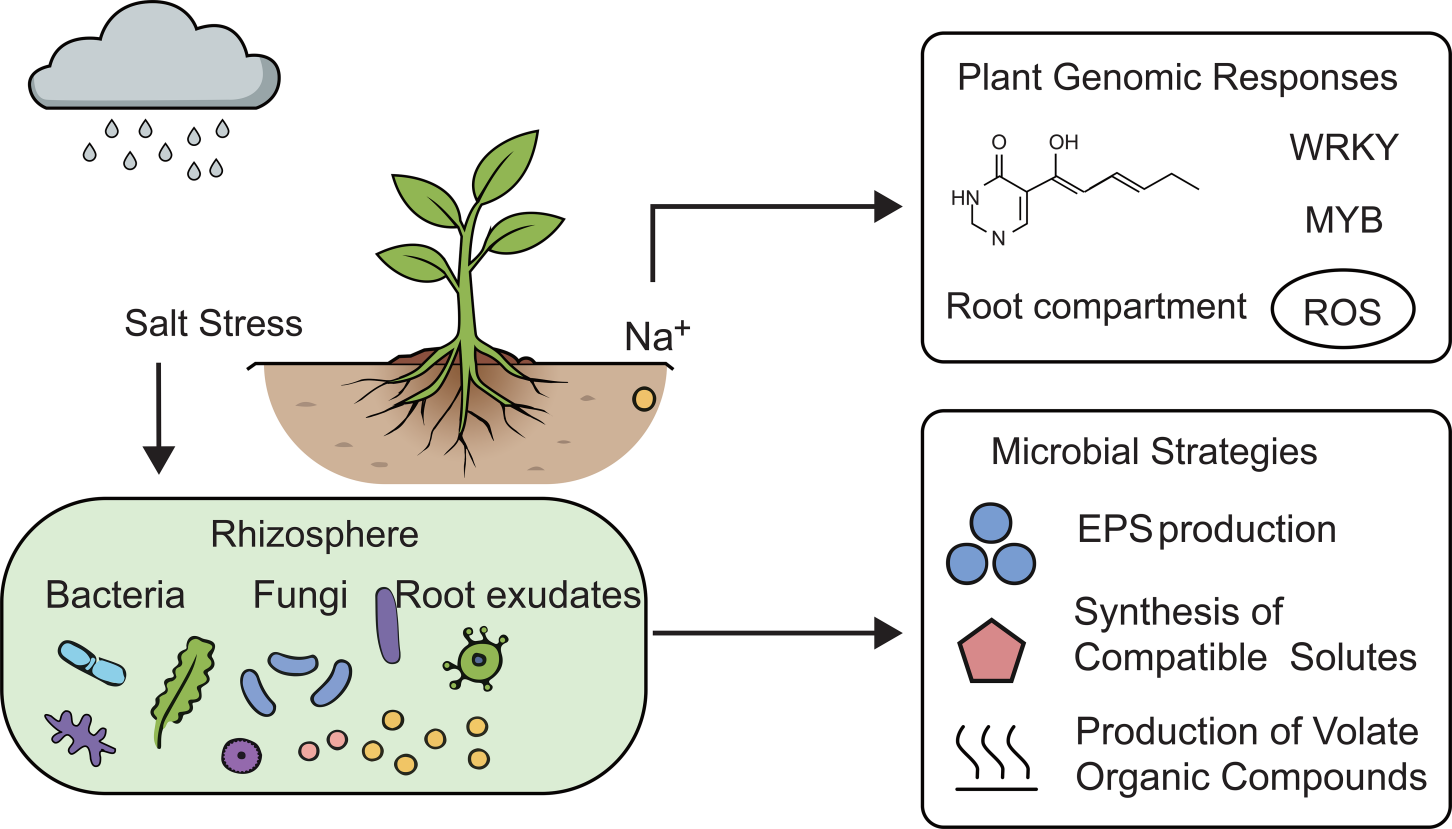
Plant–microbe genomic interplay in the rhizosphere under salt stress (original figure created by the authors).

**Figure 1** shows the plant–microbe genomic interplay and interaction in the rhizosphere under salt stress. This interaction between plant functional genes and rhizosphere microbes is vital for the mitigation of salt stress. This beneficial interaction can be used by synthetic biology, microbial inoculants, and plant breeding strategies (Gonçalves et al., 2023). Integration of plant genomics with microbial systems provides a pathway to sustainable agriculture under high soil salinity condition (Shang et al., 2024).

In addition to the adapted mechanisms of plants against salinity, over the years, there have been several conventional solutions developed against salinization such as irrigation management, gypsum application, and salt-tolerant crop breeding. However, these methods have limited effectiveness due to their slow development cycles and high input costs. Consequently, integration of microbial solutions provides an ecologically sound alternative. Microbial-based strategies have demonstrated potential for the restoration of soil health, enhancement of plant resilience, and maintenance of crop yields under salt stress (Zeng et al., 2024; Zheng et al., 2021; Neelam and Tabassum, 2023; Wu et al., 2025). 

**Figure 1** illustrates root exudate release (sugars, amino acids, phenolics) and their role in recruiting beneficial halotolerant microbes (e.g., *Sphingomonas*, *Streptomyces*). It also highlights microbial mechanisms (ACC deaminase activity, ion regulation, osmoprotectant synthesis) that improve plant Na^+^/K^+^ homeostasis, osmotic adjustment, and ROS detoxification.

Although our focus is salinity, several genomic and microbiome-mediated responses (e.g., osmotic adjustment, ROS detoxification, and transcriptional reprogramming) are shared with drought and heat; however, salinity uniquely adds ionic toxicity and the necessity for ion transport and compartmentalization. In drought conditions, plants and its associated microbiomes activate osmotic adjustment, ROS detoxification, and transcriptional reprogramming similar to salinity (Chai and Schachtman, 2022; Sorty et al., 2025), but without the burden of ionic toxicity. Drought often triggers deeper rooting patterns, enhanced abscisic acid (ABA) signaling, and the accumulation of compatible solutes such as proline to maintain water potential (Ikram et al., 2025; Ma et al., 2022). Heat stress also induces ROS scavenging and upregulates protective chaperones such as heat shock proteins (HSPs) to maintain protein stability (Hasanuzzaman et al., 2024). However, the regulatory focus under heat stress is on membrane fluidity, protein folding, and thermal signaling cascades rather than ion compartmentalization (Zhang et al., 2023). The inclusion of these comparisons offers a more integrated framework to interpret plant–microbe interactions across environmental extremes. **Table 1** presents a summary of references used in this review based on the year of publications, origins, and interest to this review.

**Table 1:** A summary of references used in this review.

Section	Number. of references	Years of publication	Countries of publication	Interest to this review paper
Introduction	12	2022–2025	China, USA, South Africa	Establishes core context of salt stress and Rhizosphere relevance
Rhizosphere as a dynamic interface	33	2018–2025	China, India, South Africa, USA, Brazil, UK, Mexico	Explores how the Rhizosphere structure influences microbial recruitment for nutrient uptake and stress resilience
Genomic perspective on plant responses	30	2021–2025	Tunisia, USA, China, Germany, Australia, Pakistan, Netherlands	Explains plant genomic mechanisms and describes root morphology for salt adaptation for better stress tolerance and shows functional gene activation in microbial partners
Rhizosphere microbial dynamics	20	2023–2025	China, India, Brazil, Portugal, Japan	Describes dynamic microbial community changes under salinity and analyzes halophyte-associated microbes for stress tolerance
Plant–microbe interactions	16	2023–2025	Italy, USA, India, China, Pakistan,	Presents signaling interactions and transcriptomic effects
Genomic and microbial synergy	16	2024–2025	South Africa, China, Tunisia, Spain,	Integrates plant genomics with microbial ecology for resilience

## 2 Rhizosphere as a dynamic interface between plant and microbial communities

**Figure 2:**
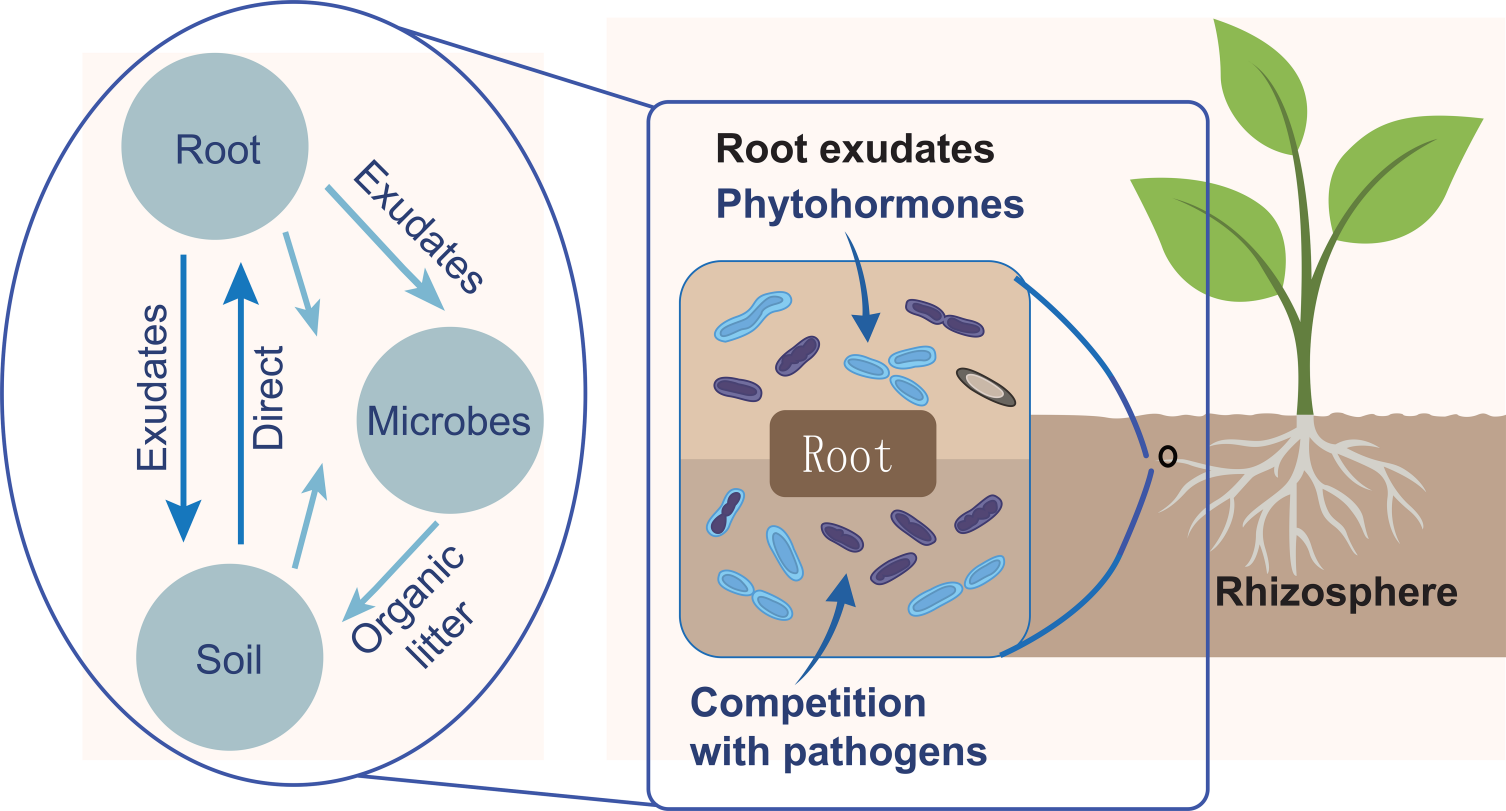
Rhizosphere interplay between soil, roots and microorganisms. Key plant genomic pathways activated under soil salt stress. Arrows indicate up regulation or interaction between pathways.

The rhizosphere is the narrow zone of soil surrounding plant roots (**Figure 2a**). It is a highly active and complex interface where plant–microbe interactions are coordinated. This zone contains a wide variety of microbial communities such as bacteria, fungi, archaea, and viruses. The composition of these communities is significantly influenced by plant species, soil characteristics, and environmental conditions (Chepsergon and Moleleki, 2023; Solomon et al., 2024; Olanrewaju and Babalola, 2022). Plant roots are the primary drivers of microbial community assembly in the rhizosphere. Through the release of root exudates—complex mixtures of sugars, amino acids, organic acids, and secondary metabolites—plants provide both energy sources and chemical signals that attract beneficial microbes and deter pathogens. Root system architecture (RSA) further shapes microbial distribution by creating distinct microenvironments along different root zones. These plant-driven processes form the foundation of rhizosphere community structure, upon which other factors such as soil properties, plant genotype, and environmental conditions exert additional influence. Microbial populations in the rhizosphere are shaped by the presence and activity of plant roots. Additionally, the rhizosphere typically has more microbes than bulk soil because of the presence of root exudates (Sasse et al., 2018; Bais et al., 2006; Fadiji et al., 2023).

Plants influence the microbial structure of the rhizosphere, while microbes, in turn, influence plant growth by producing hormones, signaling molecules, and by outcompeting pathogens. These interactions can be either beneficial or detrimental. These depend on the microbial species (Berendsen et al., 2012) (**Figure 2b**). The plant–microbe–soil relationship includes nutrient exchange and microbial-driven soil modification. These have direct effects on plant physiology such as hormone signaling and defense activation (Chai and Schachtman, 2022; Sorty et al., 2025) (**Figure 2c**).

The interaction between plants and microbes in the rhizosphere is essential for plant health. This relationship is significantly dynamic and influenced by various biotic and abiotic factors. The composition of the rhizosphere microbiome develops and changes with plant age. Young and mature plants have distinct microbial communities (de la Fuente Cantó et al., 2020). Seasonal and long-term ecological changes influence the rhizosphere composition, particularly in perennial plants. This potentially increases plant resilience to environmental stress and disease over time (Duret et al., 2024; Oppenheimer-Shaanan et al., 2022).

Soil characteristics are critical in organizing the rhizosphere. Soil properties like soil pH, soil organic matter, and water content influence the microbial diversity within the rhizosphere. Tillage and fertilizer application are two of the important practices involved in farming that can disrupt rhizosphere balance. Excessive application of chemical fertilizers and pesticide inputs are particularly harmful. These can decrease microbial diversity and reduce plant health (Solanki et al., 2024). The rhizosphere is even more critical in dryland agriculture systems. In arid conditions, there are specialized bacteria in the rhizosphere that make plants resistant to extreme temperatures and water stress. These microorganisms increase water retention, improve nutrient acquisition, produce growth hormones, and stimulate root development (Maphosa et al., 2025).

Current sciences have developed a number of methods to increase the functionality of the rhizosphere. The engineering of the rhizosphere includes methods such as using microbial inoculants and modifying plant trait genetics to favor the attraction of beneficial microbes (Solanki et al., 2024). A promising approach is the breeding of plants that are more capable of hosting symbiotic microbial communities. This may lead to increased crop yields and long-term soil fertility (Orozco-Mosqueda et al., 2022; Dwivedi et al., 2025). New technologies like metagenomics and sequencing have improved our understanding of rhizosphere dynamics. Such technologies show that infected and healthy plants prefer certain microbial communities. In accordance with some studies, plants can modulate pathogenic bacteria in the rhizosphere. This indirectly promotes beneficial microbes (Xiong et al., 2020). Some of the recent developments include synthetic microbial communities (SynComs), microbial genome editing, and biochar seed coating. The methods improve beneficial microbial interactions and colonization effectiveness and ultimately enhance plant tolerance to salinity condition (Gonçalves et al., 2023; Zeng et al., 2024; Neelam and Tabassum, 2023; Zheng et al., 2021). Collectively, these studies reveal that while the rhizosphere consistently functions as a buffering zone under salinity, the magnitude and nature of its protective effects vary with soil type, plant genotype, and microbial composition. Conflicting results regarding the relative importance of exudate chemistry versus root architecture suggest a need for integrative studies combining metabolomics, root phenotyping, and microbial community analysis.

### 2.1 Role of the rhizosphere in plant health

One of the main roles of the rhizosphere is to promote nutrient absorption by plants. The microbes in the rhizosphere positively affect the plant in different ways. They support plant growth, help absorb plants nutrients uptake from the soil, and protect the plants from disease (Dwivedi et al., 2025). Some microbes in the rhizosphere make nitrogen available to plants, while other microbes increase the phosphorus and potassium availability. These types of microbes are called plant-growth-promoting microbes (PGPMs). The PGPMs are species like *Rhizobium*, *Bacillus*, and *Pseudomonas* (Maphosa et al., 2025). The rhizosphere, home to many beneficial microbes, can help plants cope with stresses like drought and salinity, in addition to improving nutrient uptake. These microbes support plant survival by producing hormones and protective compounds that help plants take up more water and grow stronger roots (Solomon et al., 2024). Some rhizosphere bacteria also produce growth hormones such as indole-3-acetic acid (IAA) and gibberellins, encouraging healthy root and shoot growth and improving overall plant health (Pantigoso et al., 2022; Luo et al., 2025).

Microbes also protect plants from pathogens by using several mechanisms. They compete with harmful microbes for space and nutrients, produce antimicrobial substances, and improve the plant’s immune system, which is known as induced systemic resistance (ISR) (Orozco-Mosqueda et al., 2022; Chepsergon and Moleleki, 2023). Another protection mechanism of microbe is the microbial-associated molecular patterns (MAMPs), which stimulate and increase plant immunity and improve plant defense systems (Olanrewaju and Babalola, 2022; Luo et al., 2025). Soils with beneficial microbes can naturally reduce plant diseases. These soils are referred to as disease-suppressive soils. When pathogens are present in the soil, the plants remain healthy due to microbial competition and inhibition of pathogen spread (Chepsergon and Moleleki, 2023; Duret et al., 2024; Solanki et al., 2024; Dwivedi et al., 2025).

Scientists have described the rhizosphere as a “second genome” for plants, which emphasizes the effect of plants on microbial partners to perform functions beyond their own genetic capabilities. Rhizosphere microbes improve nutrient absorption, reduce stress hormone, and enhance resilience in poor soils under saline environments (Olanrewaju and Babalola, 2022; Luo et al., 2025). Moreover, recent studies showed that the rhizosphere microbiome is dynamic with plant age, environmental conditions, and genotype. Different species can develop distinct microbial communities. These communities significantly influence plant growth, stress tolerance, and disease resistance (Luo et al., 2025; Sarsaiya et al., 2025).

### 2.2 Relation between rhizosphere and beneficial microbiome

As previously mentioned, the root exudates play a vital role on the microbial community (Sasse et al., 2018; de la Fuente Cantó et al., 2020). This process is known as microbial recruitment (Chepsergon and Moleleki, 2023; Dwivedi et al., 2025). Different plant species have different rhizosphere microbes. Even the same plant in different soils may attract different microbes. Natural plants usually harbor more diverse and beneficial microbes than modern agricultural crops, as modern crop breeding has reduced certain natural root traits (Dwivedi et al., 2025).

Domestication of natural plants into crop plants has changed the rhizosphere. Crops bred for high yield have sometimes lost function for the use of good and beneficial microbes. Studies showed that natural plant relatives have more beneficial microbiomes, which illustrates the influence of plant genotype on rhizosphere structure (Dwivedi et al., 2025). Mycorrhizal fungi and rhizobia bacteria are special groups in contact with roots. They form mutual partnerships with plants. Fungi help in the absorbance of phosphorus by plants, while bacteria fix nitrogen and make nitrogen available for plants. These interactions increase nutrient availability and improve plant health and soil quality (Sarsaiya et al., 2025). There are also pathogenic microbes in the rhizosphere. However, healthy microbial communities can control them and reduce their negative effects—for example, bacteria with type VI secretion systems (T6SS) can eliminate rival species, and others produce antibiotics and siderophores to inhibit pathogens (Chepsergon and Moleleki, 2023). Microbial communities also produce compounds like enzymes, siderophores, and antibiotics that improve soil fertility and boost plant immunity (Chamkhi et al., 2022; Gianfreda, 2015). These microbes support plants to be adapted to stressful environments such as drought or salinity. This adaptation is performed by producing growth regulators and promoting systemic resistance in plants (Berendsen et al., 2012; Egamberdieva et al., 2017).

Plants exude specific root-derived compounds to utilize the beneficial microbes that increase nutrient uptake, synthesize phytohormones, and control and reduce the soil-borne pathogens (Olanrewaju and Babalola, 2022; Sasse et al., 2018). These microbial partners often function as coordinated consortia and increase their positive effects on plant growth and development. However, the molecular mechanisms of these interactions are not yet fully understood. Moreover, as mentioned before, rhizosphere microbial communities show dynamic shifts in composition based on plant species, developmental stage, and environmental conditions. These influence critical processes such as nitrogen fixation, phosphorus solubilization, and abiotic stress tolerance (Luo et al., 2025; Dwivedi et al., 2025; Pantigoso et al., 2022). Additionally, symbiotic interactions with microbes such as PGPR and mycorrhizal fungi contribute to nutrient absorption, hormonal regulation, and pathogen protection, demonstrating the fundamental importance of the rhizosphere in plant growth and resilience (Bais et al., 2006).

## 3 Genomic perspective on plant responses to salt stress

Plants activate a suite of genomic responses for survival such as the salt overly sensitive (SOS) pathway and abscisic acid (ABA) signaling (Liu X. et al., 2022; Rahman et al., 2025). From a genomic perspective, drought and salinity share several regulatory hallmarks, including the activation of ABA biosynthesis genes (e.g., NCED family), ROS-detoxifying enzymes, and stress-responsive transcription factors (Ikram et al., 2025; Fan et al., 2023). However, salinity uniquely demands the operation of SOS-, NHX-, and HKT-mediated Na^+^/K^+^ homeostasis to counter ionic toxicity (Yuan et al., 2025). Drought responses more frequently involve aquaporin regulation, cuticle biosynthesis, and hormonal cross-talk between ABA and jasmonic acid for stomatal control (Zou et al., 2021). In heat stress, overlapping ROS and TF responses are coupled with heat shock factor (HSF) activation, HSP accumulation, and expression of membrane-protective proteins such as lipid desaturases. Microbial partners can influence all of these processes, though the exact regulatory nodes differ between stresses (Kang et al., 2025).

A core aspect of the plant’s genomic response to salt stress is hormonal regulation, particularly abscisic acid (ABA), which plays a crucial role in stomatal closure and stress-related gene activation—for example, in honeysuckle (*Lonicera japonica*), members of the NCED (9-cis-epoxycarotenoid dioxygenase) gene family are enhanced under salt stress, which reveal their important role in ABA-mediated signaling and oxidative stress decrease (Lei et al., 2025). Transcription factors (TFs) control downstream target genes for osmoprotectant accumulation, ROS detoxification, and ion transport (Ikram et al., 2025; Muhammad et al., 2025; Liu Q. et al., 2022). Transcriptional regulation is often combined with epigenetic modifications like histone acetylation or DNA methylation. Furthermore, small RNAs (miRNAs) have become identified as another layer of regulation under salt stress (Ma et al., 2022).

Genomic research has significantly enriched our understanding of how plants perceive, respond to, and adapt to saline conditions. High-end technologies such as genome-wide association studies (GWAS), transcriptomics, and CRISPR-Cas9 gene editing have enabled the identification of key salt tolerance genes as well as the regulatory pathways and stress-responsive molecular mechanisms (Yuan et al., 2025; Liu Q. et al., 2022). These genomic tools are increasingly used in crop improvement programs via strategies such as marker-assisted selection. Studies indicate consistently that salt stress increases the activity of genes involved in ion transport, water balance, hormone signals, and antioxidant protection (Rehman et al., 2025; Liu X. et al., 2022). Importantly, recent research shows the interaction between plant genomic responses and rhizosphere microbial interactions for the enhancement of salt tolerance—for instance, GWAS and transcriptomic analyses identified the *FtAUR3* gene in Tartary buckwheat, which increases antioxidant capacity and redox homeostasis by regulating flavonoid biosynthesis under salinity stress (Lu et al., 2025).

In addition to this, genes such as SOS1 (Salt Overly Sensitive 1), NHX1 (Na^+^/H^+^ antiporter 1), and HKT1 (High-Affinity K^+^ Transporter 1) have been highlighted as critical for ion regulation under salinity, which offer novel alleles for breeding salt-resilient cultivars (Yuan et al., 2025). Marker-assisted selection and genome editing are increasingly promoted to accelerate the integration of genes and ion interaction under the salt stress (Rehman et al., 2025). A metabolomic perspective has also highlighted the role of flavonoid pathways with the IhCHS1 gene in *Iris halophila*, which improved tolerance via proline and JA accumulation (Liu Q. et al., 2025).

It is worth mentioning that microbial symbionts have been identified to modulate plant gene expression under stress—for example, in tomato, inoculation with *Enterobacter* sp. SA187 significantly enhanced SOS and NHX gene families and boosted antioxidant enzymes. This increases the plant tolerance to both salinity and heat (Rahman et al., 2025). Similarly, biochar-coated seeds with *Serratia nematodiphila* in maize increased the photosynthetic performance and osmotic regulation and promoted stress-responsive gene expression (Cheng et al., 2025). Root architecture was also shown to be highly adaptive under salinity with changes in morphology and exudate profiles. *Bacillus velezensis* KB21 controlled ABA and gibberellin pathways, which highlight the integrated roles of microbes and root architecture in stress resilience (Kang et al., 2025; Cheng et al., 2025). Across diverse crops, the activation of ion transporters (SOS, NHX, HKT) and transcriptional regulators (DREB, NAC, WRKY) emerges as a central genomic strategy under salinity. However, discrepancies in reported gene expression patterns—particularly under combined stress scenarios—highlight the influence of experimental conditions and genotype-specific regulation. Future research should prioritize comparative transcriptomic and functional validation studies under field-relevant, multi-stress environments.

### 3.1 Key genes and regulatory networks

Salt stress tolerance gene in plants is controlled by a complex genetic network, structural genes, transcription factors, and key signaling components. These genes work to coordinate cellular processes that maintain ionic balance, osmotic stability, and redox homeostasis under saline conditions. A well-characterized group is ion transporter genes, such as encoding Na^+^/H^+^ antiporter and K^+^ channel, which control the flux of sodium and potassium ions across cellular membranes to prevent toxicity and maintain physiological functions (Liu Q. et al., 2022; Rehman et al., 2025; Zou et al., 2021). These transporters are frequently activated as part of broader salt stress signaling pathways, which involve hormonal cues and secondary messengers.

As mentioned before, genes such as SOS1 (Salt Overly Sensitive 1), NHX1 (Na^+^/H^+^ antiporter 1), and HKT1 (High-Affinity K^+^ Transporter 1) are critical for ion regulation under salinity. The SOS1 gene encodes a plasma membrane Na^+^/H^+^ antiporter, which is involved in Na^+^ efflux from root cells. NHX1 and NHX2 encode vacuolar Na^+^/H^+^ exchangers that sequester Na^+^ into vacuoles, which reduces its toxicity in the cytoplasm (Rehman et al., 2025; Muhammad et al., 2025). The HKT1;5 gene, for example, in wheat and rice, has an important role in extracting Na^+^ from the xylem to prevent its accumulation in aerial tissues (Gudi et al., 2025)—for example, wheat lines carrying superior haplotypes of TaHKT1;5-D and TaNHX1 show better Na^+^ exclusion and higher K^+^ retention, which results in improved plant salt tolerance (Gudi et al., 2025). Similarly, tomato plants colonized by SA187 show an enhanced expression of SOS2, NHX3, and HSPs (heat shock proteins), resulting in better ion control and thermal resilience (Rahman et al., 2025).

Genes involved in the synthesis of compatible solutes such as proline and glycine betaine play critical roles. P5CS (Δ1-pyrroline-5-carboxylate synthetase) is essential for proline biosynthesis and is controlled under salt and drought conditions. These osmoprotectants stabilize proteins, membranes, and enzyme functions under stress (Ikram et al., 2025). ABA biosynthesis and signaling are core in plant salt stress responses. The NCED gene (9-cis-epoxycarotenoid dioxygenase) family regulates the rate-limiting step in ABA biosynthesis. In honeysuckle, the expression of LjNCED2 and LjNCED4 is strongly influenced by salinity, which correlated with increased stress adaptation through ABA accumulation and ROS detoxification (Lei et al., 2025). DREB (dehydration-responsive element binding), NAC, MYB, and WRKY TFs bind to stress-responsive elements in the promoter regions of key stress genes. WRKY refers to a large family of transcription factors in plants that controls gene expression, especially in response to stress and during development—for instance, WRKY and MYB TFs (growth, development, metabolism, and stress responses) control antioxidant enzyme expression, while DREB TFs (dehydration-responsive element binding proteins) activate LEA (late embryogenesis abundant) and HSP genes (Ikram et al., 2025).

### 3.2 Root architecture under salt stress

The root system, the first plant organ to encounter salinity, plays a crucial role in the adaption to saline soils through changes in root architecture and root exudation patterns (Zou et al., 2021). Salt leads to stunted root growth, reduced branching, and smaller root area. In addition, salt stress impacts primary root elongation, reduces lateral root density, and alters root angle. Gene families like SOS and HKT are strongly expressed in the root to maintain Na^+^/K^+^ balance (Ma et al., 2022). Root system architecture (RSA) describes the spatial configuration of roots in the soil. It is also a plastic trait responsive to environmental stress conditions. Under salt stress in soils, RSA is changed in multiple ways such as primary root growth, lateral root formation, root angle, and branching pattern to optimize water and nutrient uptake (Zou et al., 2021; van Zelm et al., 2023)—for instance, plants like *Arabidopsis thaliana* perform a multi-phase root growth response under salt stress, which involves a temporary growth arrest followed by recovery and adaptation to a new growth steady state (van Zelm et al., 2023). These RSA changes under salt stress are controlled by hormonal pathways. However, under high salinity conditions, auxin-dependent pathways can be removed, and alternative mechanisms take place. Zhang et al. (2023) reported that LBD16 (a gene crucial for lateral root development) remains active under salt stress via an auxin-independent pathway, which involves the transcription factor ZAT6 (zinc finger of *Arabidopsis thaliana* 6). This dual regulation allows plants to maintain lateral root branching under adverse conditions. Crop species show variable RSA responses. Shelden and Munns (2023) described that cereals such as wheat, rice, maize, and barley show different degrees of salt tolerance; however, barley appears relatively more tolerant than rice. Some breeding strategies now focus on “steep, deep, and cheap” root ideotypes to improve water and nitrogen acquisition under salt-affected soils.

Under drought, similar increases in organic acid and amino acid exudation have been reported, which help recruit microbes capable of enhancing water uptake, producing osmoprotectants, and solubilizing nutrients in dry soils (Sorty et al., 2025). Flavonoids and phenolic compounds are also commonly exuded to signal symbiotic microbes such as mycorrhizal fungi. Heat stress, though less studied in this regard, often promotes the release of phenolics, terpenoids, and specific VOCs that enhance antioxidant activity and stabilize cellular membranes (Hasanuzzaman et al., 2024). In contrast, the VOC-mediated enrichment of halotolerant taxa—such as *Sphingomonas* and *Streptomyces*—appears more characteristic of saline soils than of drought or heat (Xu et al., 2025). These differences suggest that while some exudate-mediated recruitment strategies are conserved, others are fine-tuned to match the specific stress environment.

In terms of anatomical level, salinity reduces root diameter and xylem vessel size, which are adaptations to limit water loss and control ion transport. Loudari et al. (2022) showed that phosphorus nutrition can partly alleviate salt-induced anatomical changes in wheat roots. These increase root surface area, diameter, and vascular cylinder size that increase root absorptive capacity under salinity. Shelden and Munns (2023) highlighted the utility of modern phenotyping tools (such as X-ray CT and GLO-Roots) for the assessment of RSA changes in soil. They found that the combination of these tools with transcriptomic and metabolomic profiling can identify salt-responsive plants to breed. RSA plasticity, which is defined as the ability to adapt root structure under stress, is a valuable factor for the development of salt-tolerant varieties. However, RSA plasticity and exudation are also limited by energy costs. Optimizing the balance between costs and RSA plasticity and exudation is essential for the improvement of salt tolerance without compromising the yield of crops.

### 3.3 Root exudation under salt stress

Root exudation is another important key mechanism for the adaptation of plant to salinity conditions. As mentioned, root exudates include organic acids, sugars, amino acids, and secondary metabolites released into the rhizosphere (Chai and Schachtman, 2022). Salinity develops and determines distinct root exudate profiles, often enriched in osmo-protective compounds and signaling molecules. Root exudates like sugars, amino acids, and flavonoids can be changed under salt. These molecules help using beneficial microbes and improve the rhizosphere’s condition. In maize, for example, seed coating with *Serratia nematodiphila* and biochar has been applied to enrich Proteobacteria and increase osmotic protectants such as proline and soluble sugars (Cheng et al., 2025).

Pan et al. (2022) demonstrated that salt stress might led to increased exudation of organic acids and sugars in the halophyte *Nitraria tangutorum*, which was correlated with the application of K-strategist bacteria in the rhizosphere. These microbial communities were found to be more stable and better adapted to severe environmental conditions like salinity conditions. Thereby, they can provide sustained benefits to the plant. In contrast, the glycophyte *Beta vulgaris* was reported by Wang et al. (2021) to adopt a different strategy, which relied more heavily on modifications within the rhizosphere than on changes in root morphology.

The role of root exudates in mediating belowground signaling networks was emphasized by Sorty et al. (2025), who showed the impacts of nutrient cycling, stress resilience, and ecosystem functioning. It has been estimated that up to 40% of photosynthetically fixed carbon could be assigned to root exudation, which highlights its importance in plant adaptation. These exudates also function as biochemical signals for microbial recruitment, particularly under conditions of nutrient limitation or environmental stress like salinity, thereby establishing the foundation for beneficial plant–microbe interactions.

Further observations by Chai and Schachtman (2022) indicated that specific compounds within root exudates such as carboxylates and flavonoids can facilitate the mobilization of nutrients like phosphorus and iron and promote symbiosis with mycorrhizal fungi. Under nitrogen-limited conditions, these exudates have been shown to stimulate associations with nitrogen-fixing microbes while reducing the nitrifying bacteria and enhancing nitrogen use efficiency. It was also found that the plant microbiome responds dynamically to such changes in exudation patterns. Zhu et al. (2023) reported niche-specific microbial assembly in salt-tolerant microbiomes and highlighted the presence of distinct bacterial and fungal communities across different plant compartments. These microbial communities were significantly changed under salt stress and inoculation, with the bacterium *Halomonas lutescens* shown to improve both biomass and salt tolerance. This thereby indicated the functional relevance of these microbial shifts. Similar results were found by Tiziani et al. (2022), who investigated root exudation under heat and drought stress. They reported that stress-induced changes in exudate composition significantly changed the rhizosphere microbiome structure in maize. Although their aim was on thermal and drought stress, the mechanisms observed were suggested to be applicable to salt stress as well. They also showed that patterns directed the assembly of stress-resilient microbial communities.

In molecular terms, root-derived peptides have also been reviewed by Keerthana et al. (2025) for their contributions to stress resilience and nutrient acquisition. Peptides such as CLE, CEP, and DVL were described as modulators of root meristem activity, ion transport, and hormone signaling—for instance, CLE peptides were shown to increase root architecture under phosphate starvation, while CEPs were associated with primary root elongation during nitrogen deficiency. Although direct investigations under salt stress remain unclear and limited, these control principles are considered applicable across multiple stress conditions. Additionally, evidence presented by Lei et al. (2024) showed that salt stress in *Kandelia obovata* significantly increased the dissolved organic carbon (DOC), ammonium (NH_4_^+^), and nitrate (NO_3_^−^) concentrations within the rhizosphere. These shifts contributed to the stabilization of organic matter and facilitated iron–organic carbon (Fe–OC) complexation. These thereby improve nutrient bioavailability. Changes in the carbon-to-nitrogen (C/N) ratio of root exudates were also found to selectively enrich microbial populations adapted to high-salinity environments. This reinforced the concept that exudate composition is an adaptive plant response to soil salinity.

Similarly, root exudate profiles in maize and wheat changed under salt stress. The changes are particularly in the relative abundance of sugars, organic acids, and hormones like abscisic acid and salicylic acid. These shifts and changes influenced microbial diversity and increased the presence of osmoprotective compounds such as kynurenic acid and gamma-aminobutyric acid, which can increase ion transport and nutrient uptake (Wang et al., 2021). Ion homeostasis in wheat and other crops under salinity is largely mediated by salt overly sensitive (SOS), Na^+^/H^+^ antiporter (NHX), and high-affinity K^+^ transporter (HKT) family genes (see Section 3.1 for details), which work in coordination with transcription factors such as WRKY (named after the conserved WRKY amino acid sequence), MYB (myeloblastosis family transcription factors), and NAC (NAM, ATAF1/2, and CUC2 domain-containing transcription factors). Transcription factors like WRKY, MYB, and NAC control these ion transporter genes and modulate stress signaling (Yuan et al., 2025). The hormone ABA has an important impact on salt stress. Genes like NCED1 (biosynthesis) and CYP707A1 (catabolism) change ABA levels under stress. In cucumber, for instance, inoculation with *Bacillus velezensis* increases ABA-related genes and antioxidant enzymes like catalase and superoxide dismutase (Kang et al., 2025). Recent research also shows that microbes can influence the plant signaling pathways. *Enterobacter* sp. SA187, for example, boosts the expression of SOS2, SOS4, and antioxidant genes in tomato under salt and heat stress (Rahman et al., 2025). These microbes modulate and control the gene expression by interacting with hormonal and redox signaling networks.

Similar to drought stress, salt stress induces osmotic imbalance yet differs in the additional challenge of ionic toxicity, requiring specialized ion transport and compartmentalization mechanisms. In contrast, heat stress shares some ROS detoxification and transcription factor activation patterns but lacks the ion regulation component central to salinity responses.

### 3.4 Functional gene enrichment and enzyme activity

Metagenomic analysis has shown that microbial inoculation improves rhizosphere functional gene pools, particularly those related to C, N, and P cycling. Functional gene families involved in nutrient mobilization correlate with changes in plant gene expression related to nutrient uptake pathways (Liang et al., 2025)—for instance, genes encoding nitrate transporters, phosphate carriers, and iron homeostasis regulators are frequently enhanced in plants grown with PGPR compared to untreated controls. Similarly, studies in *Hordeum marinum* treated with *Bacillus pumilus* showed controls of genes linked to osmotic regulation and antioxidative response, which reinforce plant stress tolerance through gene reprogramming (Metoui-Ben Mahmoud et al., 2025).

## 4 Rhizosphere microbial dynamics under salt stress

Salt stress changes root exudation, which attracts beneficial microbes such as *Bacillus*, *Pseudomonas*, *Sphingomonas*, and *Streptomyces*, which provide more hormone production, nutrient solubilization, and ion regulation (Xu et al., 2025; Cheng et al., 2025). The use of plant-growth-promoting rhizobacteria (PGPR) has been shown to enhance salt tolerance by reshaping the microbial community and reducing salt-sensitive pathogens like *Alternaria* while improving photosynthesis, Na^+^/K^+^ balance, and chlorophyll content (Chen Y. et al., 2025; Xu et al., 2025). Additionally, root-associated microbes such as *Variovorax* and *Bacillus cereus* improve antioxidant activity and nutrient uptake, particularly in salt-adapted genotypes like *Pyrus betulifolia*. However, genotype-specific root exudates change the microbial utilization and plant resilience (Yang et al., 2025).

### 4.1 Microbial community shifts under salt stress

Salt stress significantly changes microbial community composition and function in both the rhizosphere and bulk soil. Many non-halophilic microorganisms are inhibited under salinity while communities of halotolerant and halophilic species increase and become enriched (Xu et al., 2025; Xavier et al., 2024). Commonly observed changes in microbes include reductions in microbial diversity and biomass and the enrichment of salt-tolerant species such as Proteobacteria, Actinobacteria, Firmicutes, and Bacteroidetes (Xavier et al., 2024; Shang et al., 2024). These microbial changes are not merely reactive. Root exudates change under salt stress, influencing microbial attraction—for example, salt-exposed roots release more amino acids, organic acids, and sugars, which alter microbial populations and boost salt-resilient microbes that increase plant nutrient uptake and detoxification pathways (Lei et al., 2024; Yang Y. et al., 2025). This dynamic attraction of microbes reflects a plant strategy to engineer a salt-tolerant microbiome, supporting microbial survival under adverse conditions.

In saline soils, a significant decline in salt-sensitive microbial species is typically observed. Under prolonged salinization conditions, a shift in microbial salt tolerance mechanisms has been reported. Initially, inorganic ion accumulation is relied upon by bacteria as a primary strategy for coping with high salinity. However, soil remediation and ion concentration reduction become the dominant adaptation mechanisms (Yao et al., 2024). In addition, through metagenomic analysis, a noticeable boost in functional nutrient cycling genes has been detected in improved saline soils. These include genes that enhance processes such as nitrogen fixation, phosphate solubilization, and organic matter degradation (Liang et al., 2025). Although microbial diversity typically declines under salt stress, there have been many demonstrations showing that the functional resilience of microbial communities can even rise at times. This is due to the stronger selection for highly specialized microorganisms that play essential roles in ecosystem processes, particularly in the cycling of carbon, nitrogen, and phosphorus (Liang et al., 2025). Yao et al. (2024) investigated the mechanisms of microbial salt tolerance before and after saline soil remediation. Their findings illustrated a definite movement away from reliance on inorganic ion accumulation and toward the use of compatible solute synthesis. This was concurrent with the dramatic changes in both microbial diversity and functional capacity. Such changes indicate that soil amendments have the ability to reprogram not only the taxonomic composition but also the functional capacity of soil microbiomes. The collective evidence supports a pattern in which halotolerant taxa such as *Halomonas*, *Sphingomonas*, and *Streptomyces* are preferentially enriched under salinity. However, contrasting results in different geographic regions indicate that local edaphic factors, and historical land use may shape microbial recruitment as much as plant genotype. Longitudinal field studies are needed to disentangle these drivers and assess the stability of beneficial microbial consortia over time.

### 4.2 Rhizosphere insights from halophytes

Studies on halophytes have provided important insights into microbial resilience. The rhizospheres of species like *Salicornia fruticosa* and *Sporobolus virginicus* support keystone microbes such as *Thermoleophilia*, *Alphaproteobacteria*, and *Clostridia*. These species are functionally associated with nitrogen cycling, chemoheterotrophy, and stress resilience (Xavier et al., 2024; Yang et al., 2025). Root exudates of halophytes selectively and significantly boost bacterial communities, which are capable of osmo-protection, ion sequestration, and antioxidant activity (Xavier et al., 2024; Yang et al., 2025). These adaptations not only benefit the plant but also contribute to the ecological function of saline soils, which provide the potential to utilize halophyte-associated microbes as bioinoculants in crop systems.

### 4.3 Mechanisms of microbial salt tolerance

Microorganisms in saline environments have been found to use a range of adaptive mechanisms under salt stress. These strategies are physiological, biochemical, and genetic modifications, which improve tolerance to osmotic pressure, ion toxicity, and oxidative damage. One of the primary microbial adaptations is the accumulation of compatible solutes, such as sucrose, glycine betaine, and glucosylglycerol. These molecules are used to balance osmotic pressure (Zhu et al., 2025). They also protect vital cellular components, which include enzymes and DNA from denaturation under high salinity conditions. The regulation of ion transport is another critical mechanism. As mentioned before, ion channels and membrane-associated pumps are used by microorganisms to remove toxic Na^+^ ions or to sequester them within vacuoles (Liang et al., 2025). The main role in this process is attributed to Na^+^/H^+^ antiporters and ATPases. Furthermore, the production of exopolysaccharides (EPS) is generally observed in salt-tolerant microbes. These EPS contribute to protective biofilm formation, which promotes surface attachment and moisture retention. By binding sodium ions, EPS reduce their free concentration in the soil solution, thereby reducing salt-induced stress in both microbial communities and associated plants (Metoui-Ben Mahmoud et al., 2024). Antioxidant defense systems are employed to reduce oxidative stress, which include key enzymes such as superoxide dismutase (SOD) and catalase (CAT). These defense systems neutralize reactive oxygen species (ROS) generated under saline conditions (Fan et al., 2025).

Plant-growth-promoting rhizobacteria (PGPR) are known to be significant microbial associations that mitigate salt stress in agroecosystems. PGPR ensure plant health through various functional modes, including phytohormone biosynthesis (e.g., auxins and gibberellins), stress hormone regulation—particularly ethylene via ACC deaminase activity—activation of nutrient uptake, and ion homeostasis regulation (Chen Y. et al., 2025; Cheng et al., 2025). The role of PGPR in alleviating salinity-induced injury has been supported in different crop plants. In the study by Xu et al. (2025), it was demonstrated that PGPR reduced the effect of salt stress in maize. Salinity reduced net photosynthetic activity, chlorophyll content, potassium level, and yield but caused excessive Na^+^ uptake and ionic imbalance. Nevertheless, when PGPR were applied, photosynthetic activity, chlorophyll content, and ion homeostasis increased significantly, and loss of yield was mitigated. In addition, the rhizosphere microbiome was reorganized with increased abundance of beneficial genera such as *Streptomyces* and *Sphingomonas* and a corresponding reduction of disease-inducing taxa such as *Alternaria* and *Tausonia*.

Similar findings were reported by Chen Z. et al. (2025), who showed that rice grown under saline conditions experienced increased Na^+^ accumulation and decreased leaf water potential along with decreased yield. These adverse effects were significantly reduced by PGPR inoculation, which increased antioxidant enzyme activity and improved photosynthetic traits. This treatment reduced sodium uptake and promoted microbial diversity within the rhizosphere. Their results confirmed that PGPR contribute to salt tolerance by controlling physiological responses and restructuring microbial communities.

In rice, PGPR treatment also improved stomatal conductance, antioxidant activity, and photosynthetic efficiency (Chen Z. et al., 2025). These improvements were closely associated with changes in rhizosphere microbial composition, with an increase in beneficial bacterial- and fungal-resilient species. Further evidence was provided by Metoui-Ben Mahmoud et al. (2024), who reported that maize plants inoculated with *Bacillus pumilus* isolated from saline rhizospheres showed an increase in dry biomass, potassium uptake, and antioxidant enzyme activity under salt stress. Comparable effects have also been documented in tomato and grape systems. The combined application of PGPR and biochar was reported to restructure the rhizosphere microbial community, reduce soil salinity, and improve overall nutrient status (Li et al., 2025a; Chen Y. et al., 2025).

### 4.4 The role of the rhizosphere in enhancing plant salt-stress resilience

During the stress of salt, the rhizosphere becomes even more important, acting as a buffer zone that helps the plants reduce salt and ionic stress, maintain nutritional regeneration, and activate defense systems (chain by al., 2023a; Fu et al., 2025). Plants rely on this microenvironment quality to promote favorable microbial interactions that regulate ion homeostasis, increase the antioxidant defense, and improve the general stress tolerance.

**Figure 3:**
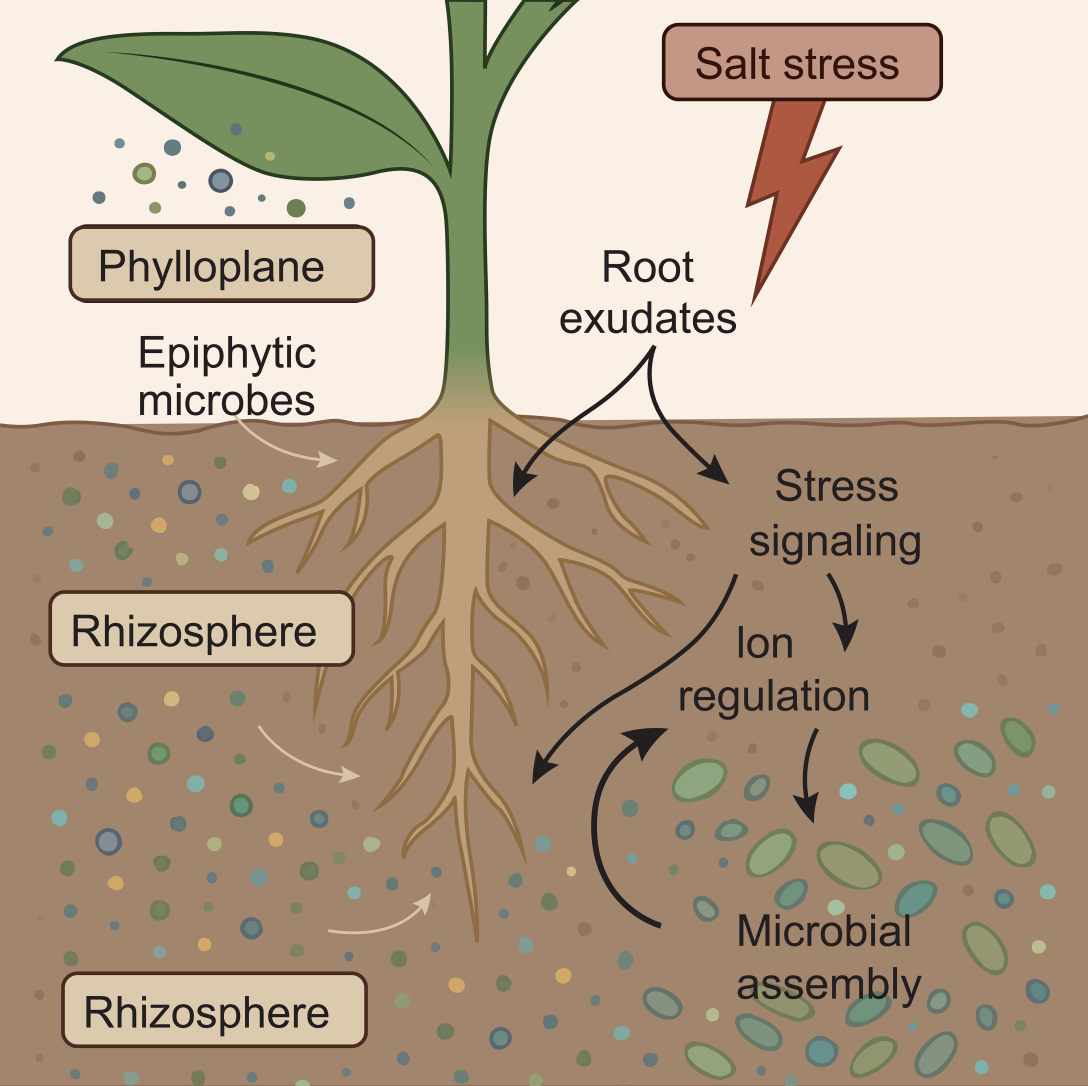
Microbes’ activities in the rhizosphere under salt stress (original figure created by the authors).

**Figure 3** shows changes in root exudate composition (organic acids, amino acids, flavonoids, VOCs) under salt stress compared with non-stress conditions, describing their selective recruitment of beneficial microbes and suppression of pathogens. Microbes in the rhizosphere live passively—they actively help shape the surroundings. Through niche discrimination, various root-coupled areas house unique microbial communities (as shown in **Figure 3**)—for example, in salt-tolerant party week species, it has been shown that room-specific microbiota affects salt flexibility (Zhu et al., 2023). Root exudates act as chemical signals that recruit or repay specific microbial treasures and form the rhizosphere composition in response to salt stress. As a result, microbial mounting plant can modify physiology and gene expression so that the plant’s capacity can cope with stress. These exudates are enriched with phenoli under signaling molecules, amino acids, organic acids, and salinity, and thus additional brine interactions are strengthened.

Rhizosphere microbial communities support systemic stress resistance by priming transcriptional regulators and adjusting signaling networks that control metabolism and cellular homeostasis (Liang et al., 2025)—for example, beneficial microbes can stimulate hormonal pathways and antioxidant responses that are key to maintaining cell integrity under salt-induced oxidative stress. Additionally, rhizosphere utilization by microbes can influence long-term soil health. Microbial changes are developed by PGPR and mycorrhizal fungi, which improve nutrient cycling and soil fertility. These contribute to sustainable crop performance in saline soils. This result highlights the importance of the rhizosphere not only as a physical zone but as a functional partner in plant adaptation to salinity.

## 5 Plant–microbe interactions under salt stress

As mentioned before, salt stress negatively impacts plant growth by increasing osmotic stress, ionic imbalance, and oxidative damage. Increasing evidence shows that microbial symbionts (particularly PGPR), endophytes, and arbuscular mycorrhizal fungi (AMF)) have critical roles in controlling these responses and improving plant salt tolerance. Evidence converges on a model where plant-associated microbes enhance salinity tolerance via a multi-tiered approach—ion regulation, osmotic balance, ROS detoxification, and hormonal modulation. Nonetheless, variation in effect sizes across studies suggests that microbe–plant compatibility, inoculation method, and environmental context are critical determinants of success. Addressing these variables in large-scale, multi-location trials will be essential to translate laboratory findings into agricultural practice.

### 5.1 Microbes modulate plant genomic responses

Recent genomic and transcriptomic studies have demonstrated that microbial inoculation induces stress-perception-, signal-transduction-, and defense-expressing genes in plants. PGPR regulate hormone-signaling pathways such as those of auxin, abscisic acid (ABA), salicylic acid (SA), and jasmonic acid (JA) and regulate gene expression to regulate osmotic adjustment, ion transport, and antioxidant defense (Giannelli et al., 2023). In maize, PGPR treatments stimulated the activity of genes related to photosynthesis, chlorophyll biosynthesis, ion homeostasis (Na^+^/K^+^ ratio), and antioxidative enzyme activity, such as peroxidases and superoxide dismutases (Xu et al., 2025). Similarly, in cucumber, *Bacillus velezensis* inoculation led to an increase in the antioxidant enzyme levels and boosted the ion balance by modulating transcription factors (e.g., MYB and WRKY), which trigger salt tolerance genes (Kang et al., 2025).

Microbial influences also affect the plant’s epigenetic landscape. The changes are not always directly measured. However, the changes in microbial abundance have contributed to changed methylation patterns, chromatin structure, and differential expression of genes related to nitrogen uptake and cell wall integrity (Wu et al., 2025)—for example, AMF induces flavonoid and alkaloid production by elevating the expression of secondary metabolism genes, increasing ROS detoxification and antioxidant capacity (Ikram et al., 2025). Microbially regulated plants modify their root exudation patterns. These exudates—regulated by organic acids, amino acids, flavonoids, and benzenoids—are both nutritional and regulatory molecules influencing root and shoot gene transcription. Gamma-aminobutyric acid (GABA), for instance, controls ion transport and root development genes.

The ability of microbial partners to modulate plant genomic responses extends beyond salinity. Under drought, similar plant–microbe communication often enhances ABA biosynthesis, upregulates aquaporins to facilitate water transport, and increases the production of extracellular polysaccharides that improve soil water retention (Ikram et al., 2025). In heat stress, microbes can induce HSP expression, enhance antioxidant systems, and contribute metabolites that stabilize membranes under elevated temperatures (Hasanuzzaman et al., 2024). These cross-stress parallels imply that certain microbe-induced transcriptional programs, particularly those related to oxidative stress mitigation and hormone regulation, may form a shared core of the plant abiotic stress response. Nevertheless, stress-specific signatures—such as ionic regulation in salinity or protein folding in heat—remain critical for full adaptation.

### 5.2 Influence of microbial volatiles and exudates

Microbial volatile organic compounds (VOCs) are considered as important mediators in plant–microbe communication and microbe–microbe communication. *Streptomyces* and *Sphingomonas* carry VOCs that are associated with upregulation increase in genes controlling systemic acquired resistance (SAR) and root elongation (Hasanuzzaman et al., 2024). For maize, PGPR treatment boosted VOCs such as benzenoids, ketones, and dibutyl phthalate. These were highly significantly correlated with the increased rhizosphere diversity along with plant biomass (Xu et al., 2025). These VOCs can also improve physiological traits such as Na^+^/K^+^ ratio and root elongation and overexpressed genes involved in photosynthesis and ion regulation. Under pear cultivar comparison, salt-tolerant ‘Qingzhen V111’ released more varied VOCs (lipids and benzenoids) than the sensitive ‘QAUP-1’, as was consistent with higher microbial diversity and better adaptation to saline–alkaline conditions (Wu et al., 2025). The integrated effect of microbial VOCs and root exudation with PGPR is critical for salt tolerance. In maize, *Serratia nematodiphila* inoculated by biochar-based seed coating increased the exudate and VOC emission with a positive impact. In addition, it boosted salt-tolerant resilience, antioxidant activity, proline deposition, and water use efficiency (Xu et al., 2025).

### 5.3 Microbiome engineering and agriculture

Microbiome engineering strategies are being developed as environmentally friendly methods of salt stress management. They include microbial inoculants that either mimic halophyte-associated communities or utilize the consortia of tolerant PGPR strains. Seed priming with sodium selenite (Na_4_SeO_3_) has been promising. In sugar beet, not only did it enhance salinity tolerance but it also increased microbial richness and reshaped the rhizosphere community (Liu A. et al., 2025). Similarly, *Serratia nematodiphila* in biochar-based coatings promoted plant growth and added beneficial genera like *Sphingomonas* and *Streptomyces* (Cheng et al., 2025). More studies are needed to validate the long-term field performance of these techniques.

Several PGPR strains, including *Bacillus pumilus*, have consistently improved biomass, K^+^ uptake, and antioxidant activity in multiple crops under salinity (Metoui-Ben Mahmoud et al., 2024). In rice, PGPR inoculation enhanced the photosynthetic activity and stomatal conductance and reduced Na^+^ accumulation while promoting microbial diversity in the rhizosphere (Chen et al., 2025). These effects have been replicated in other crops. In tomato and grape systems, the combination of PGPR with biochar not only improved nutrient profiles and reduced soil salinity but also reshaped the rhizosphere microbial structure (Li et al., 2025b; Chen Y. et al., 2025). Endophytes and AMF also induce plant tolerance mechanisms through chemical signaling and gene expression regulation (Ikram et al., 2025), echoing their central role in microbiome-based agricultural innovation.

### 5.4 The role of microbial communities in enhancing plant salt stress resilience

These mechanisms, detailed in Section 4.3, collectively enhance osmotic adjustment, ion homeostasis, and oxidative stress mitigation, with endophytic fungi such as *Fusarium equiseti* further boosting antioxidant activity and rhizosphere balance (Chen et al., 2023). Microbial communities demonstrate remarkable flexibility under saline conditions. Salinity causes significant changes in microbial diversity and composition, which is often favorable for halotolerant groups like Proteobacteria and Firmicutes (Liu et al., 2024). These changes are not passive responses but form part of an active response. However, the changes are adaptive microbial strategy to buffer plants against salt-induced damage. Key microbial functions increased under salt stress are nitrogen metabolism, amino acid biosynthesis, and antioxidant enzyme production (Fu et al., 2025). These functional changes cause the stress-induced microbial synergy, stabilize community structure, and improve plant survival. According to the stress-gradient hypothesis, facilitative microbial interactions intensify under higher environmental stress (Liu et al., 2024).

Microbes also control rhizosphere chemistry by controlling the composition of root exudates, which further affects microbiome composition. PGPR-inoculated plants secrete modified exudates that recruit positive taxa like *Streptomyces* and *Sphingomonas* that increase Na^+^/K^+^ homeostasis in plant tissue (Xu et al., 2025). Moreover, microbial inoculants can perform a “legacy effect” where, under stress, soils preconditioned with microbes carry microbial communities that deliver resilience upon later stress exposures (Yu and Zhu, 2024). Phosphorus-solubilizing bacteria (PSB) increase phosphorus supply and microbial diversity in alkaline–saline soils for the promotion of halophyte growth like *Suaeda salsa* (Sun et al., 2024).

Individual strains have failed, however, with microbial consortia being particularly valuable—for instance, mixtures of *Bacillus*, *Enterobacter*, and *Pseudomonas* initiate maize growth under salt stress through boosted enzymatic activity, nutrient uptake, and water content (Afzal et al., 2023). Biochar and nano-biochar materials support microbial colonization and improve soil structure under soil salinity condition. These amendments significantly reduce greenhouse gas emissions and increase microbial interactions, which are critical for salt tolerance (Sultan et al., 2024). In addition to soil-based applications, microbial partners provide better situation for plants to improve systemic resistance mechanisms. Species such as *Streptomyces* develop an expression of stress-related genes including those for pathogenesis-related (PR) proteins and key transcription factors (WRKY, MYB, DREB). These aid in salt and oxidative stress responses (Xu et al., 2025; Hasanuzzaman et al., 2024). In extreme environments like alpine wetlands, microbial resilience is evident of bacteria enhancement for community connectivity, while archaic change is toward stress-tolerant taxa (Liu et al., 2024). This ecological flexibility strengthens plant–microbe partnerships as viable tools for sustainable salinity management—for example, *Streptomyces* has been shown to increase the expression of genes encoding pathogenesis-related (PR) proteins and transcription factors like WRKY, MYB, and DREB (Xu et al., 2025; Hasanuzzaman et al., 2024). In environments under salt and oxidative stresses, microbial partners perform as buffers by increasing plant signaling networks. This involves both transcriptional and post-translational modifications that adjust metabolism and cell wall integrity for better stress adaptation (Liang et al., 2025).

## 6 Interaction of plant genomic, microbial, and rhizosphere components under salt stress

**Figure 4:**
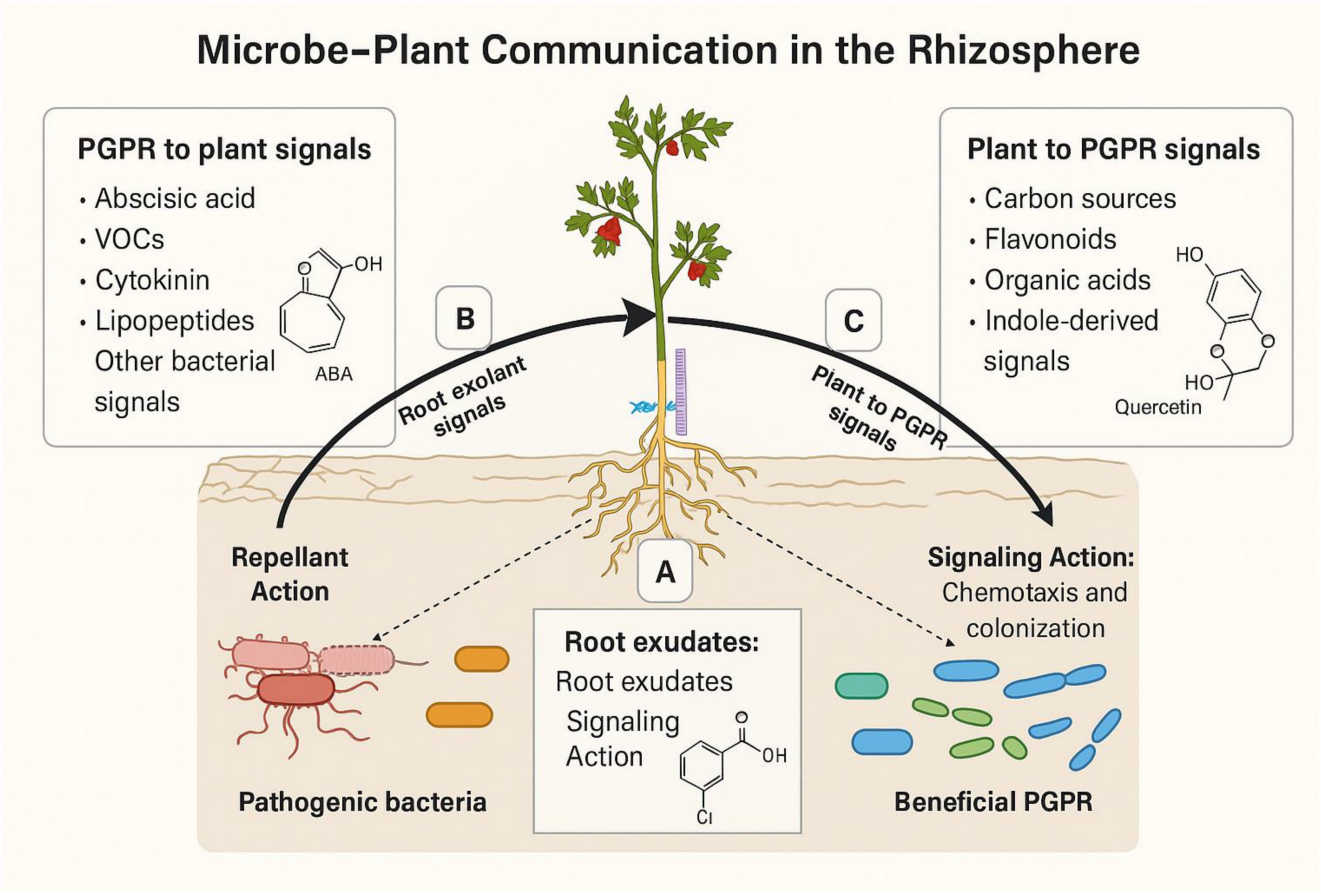
Plant, microbial, and rhizosphere interactions under salt stress (adapted from Giannelli et al. (2023)).

Soil salinity is a critical challenge, especially in arid and semi-arid regions, where climate change, poor irrigation, and land degradation exacerbate the salinity problem (Shelden and Munns, 2023; Liu et al., 2024). Salt stress weakens soil fertility, reduces microbial diversity, and significantly damages plant survival and crop productivity (Fu et al., 2025; Liang et al., 2025; Yao et al., 2024). While plants enable their own genomic defense mechanisms to respond to salt-induced stress (Zou et al., 2021; Liu X. et al., 2022; Liu A. et al., 2025), they also rely heavily on interactions with rhizosphere microbes (Berendsen et al., 2012; Xu et al., 2025). This important interplay between plant genetic regulation and microbial community dynamics has gained considerable attention for its potential in enhancing salt stress resilience (Dwivedi et al., 2025; Sliti et al., 2025) **Figure 4** shows the interplay between plant genetic regulation and microbial community rhizosphere. This figure summarizes the microbial contributions to plant salinity tolerance, including ion regulation, osmolyte production, antioxidant enzyme induction, and hormonal modulation. As shown in **Figure 4**, pathways are grouped by physiological function and linked to plant traits such as growth maintenance and yield stability under salinity.

### 6.1 Genomic and microbial synergy in plant salt tolerance

Salt stress disrupts ion homeostasis, creates osmotic imbalances, and induces oxidative stress in plants. In response, plants initiate various genomic responses, which are the expression of Na^+^/H^+^ antiporters for ion compartmentalization, antioxidant enzyme production, and synthesis of osmolytes like proline and glycine betaine (Liu Q. et al., 2022; Rehman et al., 2025; Sliti et al., 2025; Liu Q. et al., 2025). However, these certain mechanisms alone are often insufficient, especially under prolonged stress like salinity. Rhizosphere microbes, particularly PGPR such as *Azospirillum lipoferum*, *Bacillus* spp., and *Kocuria rhizophila*, play a complementary role by increasing plant stress responses. These microbes produce phytohormones (e.g., auxins), EPS, siderophores, and ACC deaminase while also improving nutrient availability (Afzal et al., 2023; Adeleke et al., 2024; Giannelli et al., 2023; Egamberdieva et al., 2017).

The influence of microbes extends to gene expression. In rice, inoculation with *Lysinibacillus fusiformis* and *Brevibacterium pityocampae* causes the enhancement of genes like *OsNHX* (for sodium exclusion), *APX* (for antioxidant defense), and *OsPIN* (for auxin transport), which increase salt tolerance (Chen Y. et al., 2025; Rahman et al., 2025). Similarly, in maize, *Pseudomonas*, *Ochrobactrum*, and *Bacillus* species improved growth and stress resistance through transcriptional modulation and metabolite regulation (Xu et al., 2025; Liu A. et al., 2025). PGPR-derived exopolysaccharides (EPS) form root-adhering biofilms that bind harmful ions and reduce osmotic stress. These permit plants to retain moisture and to absorb nutrients more effectively (Giannelli et al., 2023; Adeleke et al., 2024). Arbuscular mycorrhizal fungi (AMF) also support salt tolerance by enhancing K^+^ uptake, which regulates the Na^+^/K^+^ ratio, and start plant hormonal and antioxidant pathways (Egamberdieva et al., 2017; Chen et al., 2024; Chamkhi et al., 2022).

In saline soils, archaeal groups such as *Euryarchaeota* and *Thaumarchaeota* contribute to nutrient cycling and resilience, although their plant interactions remain less understood (Liu et al., 2024, Liu A. et al., 2025). Crucially, this genomic–microbial crosstalk is bidirectional. Under stress, plants release root exudates as “cry for help” signals, which attract beneficial microbes that activate plant genes responsible for reactive oxygen species detoxification and ion transport (Chai and Schachtman, 2022; Liu X. et al., 2022; Sorty et al., 2025)—for example, in soybean, co-inoculation with *Curtobacterium* sp. and *Bradyrhizobium japonicum* increased the expression of nodulation and stress response genes (Adeleke et al., 2024; Egamberdieva et al., 2017).

### 6.2 Rhizosphere dynamics and microbiome engineering under salt stress

The rhizosphere supports a diverse microbial community comprising bacteria, fungi, archaea, and viruses, which collectively form a “soil holobiont” that promotes plant resilience by improving nutrient cycling, reducing ionic stress, and controlling plant hormone signaling. Salt stress often induces microbial changes in the rhizosphere, which provide favorable halotolerant species like Gammaproteobacteria, Bacteroidetes, and Firmicutes, which contribute to improved antioxidant enzyme activity and activation of plant defense genes (Sliti et al., 2025). These changes are not passive but represent adaptive restructuring of the rhizosphere to buffer salinity effects.

To take advantage of these benefits, researchers are engineering synthetic microbial communities (SynComs) utilizing genome-scale metabolic networks (GSMNs). These SynComs are designed to have beneficial microbes with traits such as nitrogen fixation, IAA and GABA production, and potassium solubilization (Gonçalves et al., 2023). By ensuring metabolic compatibility, GSMNs optimize microbe–plant interactions for stress resilience. Modern tools such as metagenomics and whole genome sequencing (WGS) enable the precise identification of beneficial microbes (Dwivedi et al., 2025; Liu Q. et al., 2022)—for example, stress-tolerant rhizobacteria like *Bacillus subtilis* HAS31 have developed photosynthesis and yield in crops under both drought and salinity (Abiala, 2025). Halotolerant strains like *Enterobacter cloacae* and *Burkholderia* spp. have shown similar promise in improving plant stress tolerance (Metoui-Ben Mahmoud et al., 2025; Rahman et al., 2025). Kaya et al. (2023) reviewed hormonal and epigenetic mechanisms in plant–PGPM partnerships under drought stress. They showed that PGPMs increased drought resilience by modulating ABA, auxin, cytokinin, and ethylene signaling. Furthermore, epigenetic reprogramming via DNA methylation, histone modification, and non-coding RNAs were also considered as affecting mechanisms on drought resilience. These processes influence on osmotic adjustment, antioxidant defense, and water use efficiency—traits that also contribute to salinity tolerance. Such mechanistic overlaps strengthen the case for designing PGPM consortia with cross-protective benefits against both drought and salinity.

Recent studies showed further evidence of rhizosphere-driven molecular changes. *Bacillus* strains increase tomato salt tolerance by controlling myo-inositol metabolism and osmolyte production, while *Pseudomonas aeruginosa* HG28–5 improves Na^+^/K^+^ homeostasis through ABA-mediated signaling pathways (Chen Y. et al., 2025; Kang et al., 2025). Application of volatile fatty acids (VFAs) in saline soil has been proven to change the rhizosphere microbial composition and increase the metabolite output. These act as both substrates and signaling molecules (Chen et al., 2024; Liu Q. et al., 2025). Moreover, integrating arbuscular mycorrhizal fungi (AMF) with biochar has improved soil enzyme activity and plant biomass in saline–alkali environments, indicating the potential of combining biotic and abiotic amendments for soil and crop health (Sultan et al., 2024; Xu et al., 2025).

## 7 Future research directions

Despite the impressive advancement in understanding the role of microorganisms and biostimulants in increasing the tolerance of plants toward biotic as well as abiotic stress, there remain huge gaps in this field. Most of the reported works have been conducted mostly in controlled environments (greenhouse or lab) and then to be transferred to real-world field conditions. In addition, the precise molecular mechanisms and interactions of microbes, plants, and the environment remain to be addressed. Aspects such as plant–microorganism genetic diversity, specific responses to specific types of stresses, and stability of the performance of biostimulants under different climatic conditions are areas that require to be studied. Moreover, preparation of eco-friendly and sustainable commercial products and exploration of the long-term effect of their use on the soil ecosystem are main areas of research agenda for the coming years.

## 8 Conclusion

Salt stress is a multifaceted problem that reduces plant health, fertility of the soil, and crop productivity. This review focused on the key contribution of plant–microbe interactions to counteract salinity stress. Plants deploy an enormous arsenal of genomic solutions to deal with ionic and redox imbalances; however, these are excessively increased by the rhizosphere good microbes. These microbial partners not only contribute to nutrient acquisition and hormone regulation but also control gene expression and signal transduction networks. Root exudates act as central facilitators, which decide and modulate the microbial recruitment and feedback phenomena. Novel technologies like metagenomics, synthetic microbial consortia, and genome editing provide useful tools to identify these interactions. In the future, integrative research that unites plant genomics, microbial ecology, and soil science will be crucial for the development of resilient agroecosystems. Strategic microbiome engineering and rhizosphere management are primary tools for the enhancement of crop performance under salt stress and for attaining long-term agricultural sustainability. Integrating evidence from drought and heat stress research highlights that while many plant–microbe strategies—such as activation of antioxidant enzymes, osmolyte production, and recruitment of beneficial microbes—are broadly conserved across abiotic stresses, salinity imposes an added layer of ionic stress that requires specialized Na^+^/K^+^ transporters and compartmentalization mechanisms. In drought, the emphasis is on hydraulic adjustment, stomatal regulation, and root system architecture, while in heat stress, maintaining protein stability and membrane integrity is paramount. Recognizing both shared and unique mechanisms is essential to develop integrated breeding and microbiome engineering strategies that target multi-stress resilience in crops. Such a comparative perspective not only broadens the applicability of the current review but also identifies knowledge gaps where cross-stress lessons could accelerate innovation.

## Glossary

Rhizosphere: the narrow region of soil directly influenced by root secretions and associated microbial activity
NCED: stands for 9-cis-epoxycarotenoid dioxygenase—an important enzyme-coding gene family in plants
PGPR (plant growth-promoting rhizobacteria): beneficial bacteria in the rhizosphere that stimulate plant growth by enhancing nutrient uptake, producing hormones, or protecting against stress
AMF (arbuscular mycorrhizal fungi): symbiotic fungi that colonize plant roots, aiding in water and nutrient absorption, especially phosphorus
ABA (abscisic acid): a plant hormone involved in stress responses, particularly drought and salinity
VOCs (volatile organic compounds): small organic molecules released by plants or microbes that mediate interactions and signaling, influencing growth, defense, and microbial recruitment
Root exudates: a mixture of compounds (sugars, amino acids, and organic acids) secreted by roots that influence soil microbiota and nutrient dynamics
SOS pathway: a plant signaling pathway (salt overly sensitive) that regulates ion homeostasis under salt stress
ROS (reactive oxygen species): highly reactive molecules formed under stress that can damage cells
Compatible solutes: organic compounds (e.g., proline, glycine betaine) synthesized by plants or microbes to maintain osmotic balance under stress
Synthetic microbial communities (SynComs): man-made consortia of beneficial microbes assembled to enhance plant health and resilience
Epigenetic regulation: heritable changes in gene expression not caused by DNA sequence changes, often through methylation or histone modification
Transcriptomics: the study of all RNA transcripts produced in a cell or tissue, used to analyze gene expression patterns under stress
NHX: Na^+^/H^+^
antiporter (e.g.: NHX1, NHX2): involved in sodium sequestration into vacuoles
RSA: root system architecture
TFs: transcription factors
LEA: late embryogenesis abundant
HSP: heat shock protein
GWAS: genome-wide association studies
CRISPR: clustered regularly interspaced short palindromic repeats.
